# Cardiofaciocutaneous syndrome with rare structural variant in *DOCK8* gene associated with neurodevelopmental disorders

**DOI:** 10.1002/ccr3.2729

**Published:** 2020-02-14

**Authors:** Domenico Dell'Edera, Lucantonio Debellis, Angela Mitidieri, Annunziata Anna Epifania, Eustachio Cuscianna, Arianna Allegretti

**Affiliations:** ^1^ Unit of Cytogenetic and Molecular Genetics “Madonna delle Grazie” Hospital Matera Italy

**Keywords:** BRAF, cardiofaciocutaneous syndrome, DOCK8, multiple congenital anomaly, neurodevelopmental disorders

## Abstract

We describe a girl with clinical signs of cardiofaciocutaneous syndrome who simultaneously presents a mutation in the BRAF gene and a 9p24.3 microduplication. This genetic condition has never been described in the literature and could explain the phenotypic variability observed in the girl.

## Graphical abstract

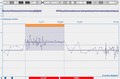

## 1 INTRODUCTION

Neurodevelopmental disorders (NDDs) are a group of diseases characterized by development brain dysfunction, such as neuropsychiatric problems or impaired motor function, learning, language, or nonverbal communication. The cause of such disorder is multifactorial.^1^

Neurodevelopmental disorders have a multifactorial pathogenesis with a relevant genetic component. Comparative genomic hybridization (A‐CGH) is the first genetic test to be performed for children with NDDs.^2^ From data in the literature, there is a genetic cause^3^, ^4^ in more than 10% of patients with autism spectrum disorders and in about 20% of children with global developmental delay. Rare copy number variations (CNVs) have been observed on chromosome 9p24.3 where DOCK8 gene is located. Most alterations are located on the first exon of DOCK8 gene. The protein encoded by DOCK8 is involved in the correct migration of immune cells.^5^, ^6^ Recently, a number of clinical trials have led to considering such CNVs as clinically relevant.^7^, ^8^

Among intellectual disabilities, there is a group of diseases called RASopathie,^9^ (cardiofaciocutaneous syndrome,^10^ Noonan syndrome). These syndromes are similar because they share the Ras/mitogen‐activated protein kinase (RAS/MAPK) pathway.^11^

cardiofaciocutaneous syndrome (CFC) is a genetic disorder with autosomal dominant transmission caused by mutations in genes BRAF,^12^ MEK1,^13^ MEK2,^14^ or rarely KRAS.^15^ In most patients with CFC, ventriculomegaly/hydrocephalus and epilepsy are present. CFC patients do not show eyebrows (ulerythema ophryogenes). Moreover, there are difficulties in feeding, gastroesophageal reflux and in about 50% of patients there are convulsions.

The most common alterations in gastrointestinal system are related to intestinal malrotation, anal stenosis, and megacolon.

We report a case of a patient showing clinical manifestations associated with the RAS/MAPK syndrome with a mutation in the BRAF gene associated with the CFC syndrome and a 315.16 kb microduplication on the short arm of chromosome 9 involving the DOCK8 and partially KANK1 genes.

The patient shows both a mutation in BRAF gene and a microduplication 9p24.3. The mother and a one of the two sisters show the mutation in BRAF gene only. The presence of both the two mutations found in the proband could explain the phenotypic difference between the patient in respect to her mother and sister.

## 2 CASE REPORT

A 18‐month‐old female girl was admitted to our unit for triangular face, external intercanthal distance of 9 cm, internal intercanthal distance of 2.5 cm, low‐set posteriorly rotated ears, micrognathia, low hairline, macrocephaly, and pectus excavatum. She was born at 38 weeks of uncomplicated pregnancy from unrelated Italian parents.

The mother was affected by mild mental retardation (MR), interatrial defect (DIA), minor dysmorphism, and hypothyroidism.

**Figure 1:**
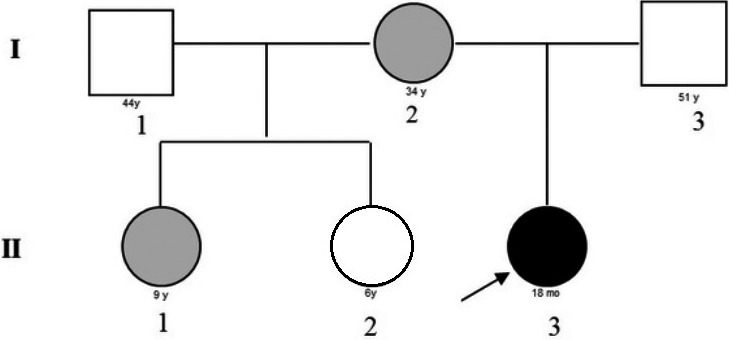
Pedigree. I2: mother (age 34 y); II1: first daughter (age 9 y); II2 second daughter (age 6 y); and II3: patient (age 18 mo). Gray: mutation in exon 6 of BRAF gene (p.Gln262Arg). Black: mutation in exon 6 of BRAF gene (p.Gln262Arg) and de novo microduplication of the short arm of chromosome 9 (9p24.3) involving DOCK8 and partially KANK1 genes

The patient (Figure 1, II3: GL) has two sisters born from the first marriage of the mother (Figure 1, II1: first daughter MB, II2: second daughter CB). The first daughter (MB) was affected by interatrial defect (DIA), while the second one (CB) showed a neurodevelopmental disorder (epilepsy, hyperactivity, and attention‐deficit). At the age of 4 months, GL was hospitalized at the Neonatology and Pediatrics Unit of Matera Hospital for sickness associated with diarrhea and weight loss.

A barium enema highlighted an intestinal malrotation (mesenterium commune). This anatomical defect was responsible for the clinical symptomatology of the proband (abdominal pain, nausea, and constipation).

The patient was affected by pulmonary valve stenosis with slight dilatation of the trunk of the pulmonary artery and persistence of the foramen ovale (PFO).

Electroencephalogram in hypnotic deprivation and quiet sleep highlighted physiologically asymmetric presence of sleep spindles, sometimes followed by slow waves. Elements of irritative significance were not appreciated. This examination was requested following the verification of two episodes of asphyxia characterized by fixity of the gaze, perioral cyanosis, generalized hypertonia, and loss of consciousness lasting 30 seconds.

Blood examination relieved an immunodeficiency of the T and B lymphocytes, characterized by recurrent skin viral infections and increased serum immunoglobulin type E (IgE) levels.

Neonatal screening for inherited metabolic disorders was negative.

On the basis of clinical evidence, a diagnosis of RASopathy was supposed.

## 3 MATERIALS AND METHODS

### 3.1 Cytogenetic analysis

Karyotype of the patient and her parents was normal.

### 3.2 DNA preparation

Genomic DNA was extracted from peripheral blood leukocytes using the commercial kit "QIAamp^®^ DSP DNA Blood Mini” (DNA IQTM System).

### 3.3 Next‐Generation Sequencing Systems

Next‐Generation Sequencing for study of RASopathies was performed on all family members (patient, two half‐sisters, mother, and father).

Parallel massive sequencing of amplicons of exons and adjacent intronic regions (±25bp) of the 14 genes (A2ML1, BRAF, CBL, HRAS, KRAS, MAP2K1, MAP2K2, NRAS, PTPN11, RAF1, RIT1, SHOC2, SOS1, and SPRED1) using the "Ion AmpliSeq" commercial panel was performed. The regions of interest were enriched by multiplex PCR (AmpliSeq Ion Torrent Technology) and analyzed by massive sequencing in parallel on Ion Gene Studio S5. Ion Reporter and Alamut software for data analysis were used with Human Reference Genome hg19, dbSNP138, HGMD as reference databases.

A c.785A > G (p. Gln262Arg) mutation was identified in exon 6 of the BRAF gene.

### 3.4 CGH array

Array‐based Comparative Genomic Hybridization (CGH) analysis was performed using commercially available microarray of oligonucleotides containing approximately 180 000 probes with an average estimated resolution of approximately 25 Kb (Oxford Gene Technology) using "CytoSure Interpret Software Version 4.10." Genomic region analysis was performed according to the human reference sequence hg19GRCh37. In summary, the copy number variations (CNVs) found in the proband were compared with genomic variants present on different databases (DECIPHER: https://decipher.sanger.ac.uk%2014UCSC Genome Browser: https://genome.ucsc.edu%2014International Standard for Cytogenomic Arrays consortium: https://www.iscaconsortium.org/index.php/search-Troina Database of Human CNVs: http://gvarianti.ho-melinux.net/gvariantib37/index.php).

## 4 RESULTS

**Figure 2:**
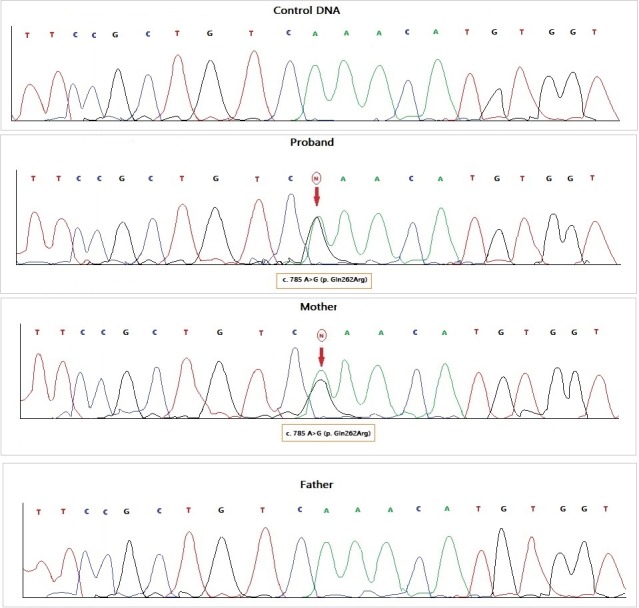
A, Localization of the mutation in BRAF gene. B, Sanger method sequencing. Red arrow indicates the c. 785 A > G (p.Gln262Arg) mutation in exon 6 of BRAF gene in the patient and her mother

Next‐Generation Sequencing analysis showed a mutation in exon 6 of the BRAF gene (locus 7q34: p. Gln262Arg) causing the CFC disease. Such mutation was inherited from the mother (Figure 2), and it is present in one of the two sisters (MB). Results obtained were confirmed by sequencing the BRAF gene with Sanger method.

**Figure 3:**
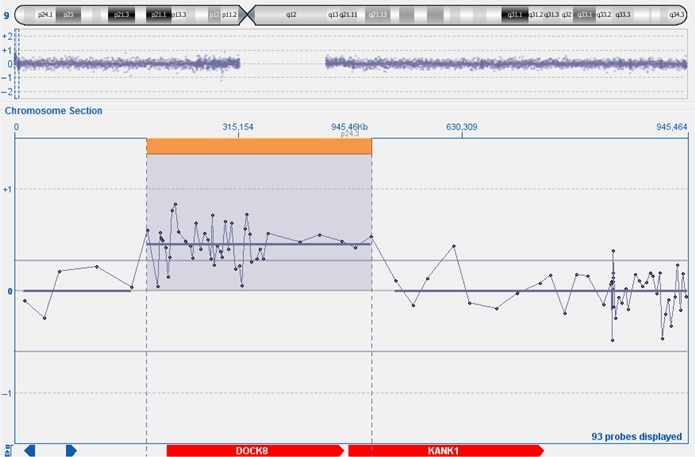
Top panel: chromosome 9 ideogram with 9p24.3 (1856610_500765) duplicated region (small orange box). Central panel: A‐CGH scatter plot showing a 315.16 Kb duplication of 9p24.3. Bottom panel: The University of California, Santa Cruz (GRCh37/hg19 assembly) genes in the overlapping region (in red)

In the proband, a de novo microduplication of 315.16 kb was detected by A‐CGH on the short arm of chromosome 9 (9p24.3) involving DOCK8 gene and partially KANK1 (Figure 3).

Mutations of these genes are reported to be involved in neurodevelopmental disorders.^16^, ^17^

Patients with similar alteration interstitial 9p24.3 involving only DOCK8 and KANK1 genes are reported. Interestingly, the patients showed nonoverlapping phenotypic traits ranging from a complex phenotype to trigonocephaly with minor dysmorphic features and hand anomalies.^8^, ^18^

The patient showed a different phenotype in respect of that one founded in her mother and sister. She was affected by immunodeficiency of the T and B lymphocytes with recurrent skin viral infections, increased serum immunoglobulin E (IgE) levels, and intestinal malrotation (mesenterium commune).

Such phenotype could be related to the presence of both the microduplication 9p24.3 and the mutation in exon 6 of the BRAF gene.

## 5 CONCLUSION

The 9p24.3 microduplication involving DOCK8 and partially KANK1 genes is associated with neurodevelopmental disorders, while the mutation in the BRAF gene is associated with CFC syndrome. Cases presenting both the 9p24.3 microduplication and the mutation in BRAF gene are not described in literature. The case presented in this report enriches the phenotypical spectrum linked to 9p23.4 microduplication associated with BRAF gene mutation.

## INFORMED CONSENT

Written informed consent was obtained from all the patients (including legal guardians of the children) for the publication of such case report and accompanying images. A copy of the written consent is available for review by the editor in chief of this journal. This study was approved by local institutional ethics committee.

## CONFLICT OF INTEREST

The authors declare that they have no competing interests.

## AUTHOR CONTRIBUTIONS

Domenico Dell’Edera: made substantial contributions to conception and design. Angela Mitidieri and Eustachio Cuscianna: contributed to the acquisition, analysis, and interpretation of data. Annunziata Anna Epifania: involved in drafting the manuscript. Arianna Allegretti: gave final approval of the version to be published. All authors: read and approved the final manuscript.
